# Synthesis of Mesoporous Zn_1−x_M_x_Al_2_O_4_ Substituted by Co^2+^ and Ni^2+^ Ions and Application in the Photodegradation of Rhodamine B

**DOI:** 10.3390/ma13092150

**Published:** 2020-05-06

**Authors:** Nilson Machado Pontes do Nascimento, Bárbara Ronara Machado de Lima, José Roberto Zamian, Carlos Emmerson Ferreira da Costa, Luís Adriano Santos do Nascimento, Rafael Luque, Geraldo Narciso da Rocha Filho

**Affiliations:** 1 Graduation Program in Chemistry, Federal University of Pará, Augusto Corrêa Street, Guamá, Belém 66075-110, PA, Brazil; nilson-pontes@hotmail.com (N.M.P.d.N.); barbararonara@hotmail.com (B.R.M.d.L.); zamian@ufpa.br (J.R.Z.); emmersoncosta@yahoo.com.br (C.E.F.d.C.); adrlui1@yahoo.com.br (L.A.S.d.N.); 2 Laboratory of Catalysis and Oilchemistry, Federal University of Pará, Street Augusto Correia, Guamá, Belém 66075-110, PA, Brazil; 3 Laboratory of Oils of the Amazon, Federal University of Pará, Perimetral Avenue, Guamá, Belém 66075-110, PA, Brazil; 4 Graduation Program in Biotechnology, Federal University of Pará, Augusto Corrêa Street, Guamá, Belém 66075-110, PA, Brazil; 5 Department of Organic Chemistry, Universidad de Córdoba, Ctra Nnal IV-A, Km 396, E14014 Cordoba, Spain; q62alsor@uco.es

**Keywords:** zinc aluminate, spinel, photocatalysis, chitosan

## Abstract

A new mesoporous Zn_1-x_M_x_Al_2_O_4_ photocatalyst was prepared using the metal-chitosan complexation method with different degrees of Zn^2+^ cation substitution with cobalt and nickel ions (M = Co^2+^ and Ni^2+^). Characterization using X-ray diffraction (XRD), Infrared absorption spectrometry (FTIR), energy dispersion spectroscopy (EDS), diffuse reflectance spectrometry (DRS), scanning electron miscoscopy (SEM), transmission electron miscroscopy (TEM), N_2_ adsorption- desorption isotherms using the Barrett-Joyner-Halenda (BJH) method, thermogravimetric analysis (TG) and differential thermal analysis (DTA) confirmed the formation of the spinel phase and high purity for all samples. N_2_ adsorption/desorption and size pore distribution confirmed the high surface area. The photocatalytic activity of Zn_1-x_M_x_Al_2_O_4_ and the effect of replacing Zn^2+^ ions with Ni^2+^ and Co^2+^ on the degradation of rhodamine B under ultraviolet light were studied in detail. The sample containing 0.1 mol of cobalt had the highest removal rate reaching 83%, favored by surface area and material bandgap (109 m^2^ g^−1^ and 2.19 eV, respectively).

## 1. Introduction

Human activities produce large amounts of contaminants including oils, greases, dyes, pesticides, among others, causing serious problems to the population, compromising the air, soil and groundwater qualities [1,2]. Industrial effluents, such as those from the textile industry, may contain organic molecules that cause environmental pollution [2]. The development of methodologies for environmental remediation has received significant attention in recent years [1]. Chen and collaborators [1] used the adsorption process for the remediation of waters, utilizing a new adsorbent system from organosilica nanoparticles with adjusted composition for the removal of anionic or cationic dyes with high adsorption capacity and fast adsorption. Liu and collaborators [3] combined the photocatalytic and adsorbent properties of core-shell Cu-BTC@TiO_2_ microspheres to improve performance in the adsorptive desulfurization of thiophenic compounds from fuels under UV irradiation. While the TiO_2_ layer on the surface of Cu-BTC promoted the photocatalytic oxidation of S thiophenic compounds, Cu-BTC adsorbed such oxidation products (sulfoxides and sulfones). The proposed desulfurization mechanism and the synergistic effect of Cu-BTC (as an adsorbent) and TiO_2_ (as a catalyst), which capture hydroxyl radicals/activate molecules, contributed to the efficient transfer of photo-excited electrons and oxidation.

In this context, semiconductor photocatalysis emerged as a technology capable of assisting in several processes, including the degradation of organic pollutants [4,5,6,7,8,9]. Compounds with crystalline structure similar to spinel have received significant attention in applications in photocatalysis, due to their semiconductor properties, and besides being non-toxic, they have high thermal stability, resistance in acid and basic media and high surface area, among other properties [10,11].

Spinels are ternary oxides of binary transition metals and may have a varied structure of general formula AB_2_O_4_, where A corresponds to bivalent cations and B to trivalent cations. In normal spinel, A^2+^ ions occupy the tetrahedral sites surrounded by four O^2−^ ions, while B^3+^ ions occupy the octahedral sites surrounded by six O^2−^ ions. The inverse spinel has the tetrahedral sites occupied by B^3+^ ions, while the octahedral sites are occupied by equal numbers of A^2+^ and B^3+^ ions with general formula (B)[AB]O_4_, where the parenthesis cations are located at the tetrahedral, and the cations in brackets are in the octahedral sites. Finally, there is the intermediate spinel of the general formula, (A_1−x_B_x_)[A_x_B_2−x_]O_4_, where x represents the conversion factor from one spinel to another [9,10,12,13,14].

Lattice enthalpy calculations based on simple ionic models indicate that for A^2+^ and B^3+^ ions the normal spinel structure is preferred, inducing greater lattice stability; however, some spinel-like structures, especially involving the block metal cations, do not meet this expectation and have been related to the stabilizing energy effect of the ligand field at the ion preference. Several spinel-like semiconductor materials have been tested in photocatalytic processes, demonstrating high activity in the photodegradation of organic pollutants [4,5,6,7,8,9,15].

For example, Hassanzadeh-Tabrizi and colleagues [16] synthesized copper aluminate, CuAl_2_O_4_, at much lower temperatures when using the co-precipitation method, also obtaining excellent results regarding the photocatalytic activities of methyl orange degradation, reaching approximately 90% in just 1 h of radiation exposure. Furthermore, in order to investigate the photodegradation of methyl orange under visible light, but this time modifying the synthesis methodology, Yanyan and colleagues [17] synthesized CuAl_2_O_4_ ysing the sol-gel method and achieved an impressive 97% degradation in 120 min of reaction.

Cobalt aluminate was also studied by Khademolhoseini and Talebi [9] as a catalyst in the degradation reaction of methyl orange under ultraviolet light, achieving 68% efficiency in 60 min of reaction.

ZnAl_2_O_4_ has received significant attention among spinel materials, being widely used as catalyst and catalyst support [11,15]. There are a variety of works developed using pure zinc aluminate in photocatalytic processes. For example, ZnAl_2_O_4_ has been applied in the degradation of pollutants including direct black 38 [15], reactive red 141 [18], phenol [15] and toluene gas [19], among others.

The structural properties of materials for catalytic purposes such as crystalline phase, crystallite size, surface area and average pore distribution are of great importance, as the increase of these properties produces greater contact of the active phase. Commercial zinc aluminate has a low surface area and low pore volume, characteristics that make contact with the material to be degraded and the photocatalyst less favorable. However, obtaining this material by different synthesis routes has significantly improved its properties, thus allowing it to be used with greater efficiency in photocatalysis [16,18,19,20,21,22].

ZnAl_2_O_4_ has a wide bandgap, making it suitable for photocatalytic applications in the ultraviolet range. However, several studies have reported that the ZnAl_2_O_4_ bandgap changes according to the synthesis method employed. Battiston et al. [10] reported a bandgap equal to 3.8 eV for ZnAl_2_O_4_ synthesized via the co-precipitation method. Anand, Kennedy and Vijaya [12] for the same material, produced using the microwave combustion method, obtained a bandgap equal to 5.01 eV. Yanyan et al. [17] produced ZnAl_2_O_4_ via the sol-gel synthesis route with bandgap values of 3.25 and 3.33 eV. In the literature, few studies have been developed and published in order to evaluate the influence that metal doping has on its final photocatalytic activity [10,23].

According to the above, this research was aimed to prepare ZnAl_2_O_4_ using the metal-chitosan complexation method with partial replacement of Zn^2+^ by Co^2+^ and Ni^2+^, producing a new mesoporous catalyst with high surface area. In addition, we investigated for the first time the photocatalytic activity of mesoporous Zn_1−x_M_x_Al_2_O_4_ synthesized using metal-chitosan complexation and the effect of replacing Zn^2+^ ions with Ni^2+^ and Co^2+^ on the degradation of rhodamine B under ultraviolet light irradiation. Ni^2+^ and Co^2+^ ions were prioritized for the replacement of zinc in the structure of ZnAl_2_O_4_, due to their periodic properties. Both ions have coordination 4 and 6, in addition to having an ionic radius similar to each other, which favors substitution.

## 2. Experimental

### 2.1. Reagents

The reagents used for the synthesis of photocatalysts present in this work were not subjected to any purification treatment and were all of analytical grade and high purity: chitosan (Sigma-Aldrich, St. Louis, MO, USA), acetic acid (99.8%, Neon, Suzano, São Paulo, Brazil), Zn(NO_3_)_2_ 6H_2_O (96%, Dinâmica, Indaiatuba, São Paulo, Brazil), Al(NO_3_)_3_ 9H_2_O (98%, Dinâmica, Indaiatuba, São Paulo, Brazil) and NH_4_OH (28–30%, Dinâmica, Indaiatuba, São Paulo, Brazil).

To obtain 2.0 g of the sample of zinc aluminate (ZnAl_2_O_4_), initially 2.5 g of chitosan were dissolved in 100 mL of acetic acid solution (5% v/v), under stirring. Then, two solutions were prepared: 3.02 g of Zn(NO_3_)_2_ 6H_2_O was dissolved in 5.08 mL of distilled water and 8.0 g of Al(NO_3_)_3_ 9H_2_O was dissolved in 7.62 mL of distilled water. Subsequently, the two solutions formed using Al and Zn were added dropwise to the chitosan solution under constant stirring, after which the mixture was stirred for 1 h. The resulting Zn-Al-chitosan solution (at a 1:2:1.5 molar ratio) was added dropwise in 50 mL, 50% v/v NH_4_OH, adjusting the pH of the solution to remain in 9.0 ± 0.5, stirring for 3 h until complete homogenization.

Spheres consisting of a chitosan complex with the metal hydroxides of Zn^2+^ and Al^3+^ were observed, filtered and washed with distilled water. They were then dried at room temperature for 48 h. They were calcined for 4 h at different temperatures (550, 650, 750, 850 and 950 °C) to eliminate residual organic matter and spinel formation. After this process, the formed product was slowly cooled to room temperature.

The synthesis of zinc aluminates substituted using Ni^2+^ and Co^2+^ metal ions followed the same methodology; however, the precursor salts of the metals were added in specific theoretical stoichiometric proportions in order to obtain the samples in the desired theoretical molar ratio [21,24,25].

### 2.2. Characterization of Synthesized Materials

X-ray diffractograms ware obtained using the D8 BRUKER (Madison, WI, USA) powder diffractometer method using CuKα radiation (0.15406 nm) at a scan rate of 0.02 °/min, the voltage and current of the tube were 40 kV and 40 mA, respectively. Infrared analysis with Fourier transform was performed using the KBr pellet method in the region of 900 to 400 cm^−1^ using BRUKER VERTEX 70 v equipment.

The morphological characteristics were evaluated using a TESCAN scanning electron microscope model VEGA3 (Fuveau, Village of Provence, France), with an electron beam current in the range of 85–90 µA, and transmission electron microscopy, with a TEM FEI Tecnai G2T20 device (Milan, Lombardy, Italy).

Surface area, diameter and pore volume were calculated according to the Brunauer-Emmett-Teller and Barret-Joyner-Halenda (BET/BJH) methods using a Micromeritics Vacprep 061 Sample Degas System (Norcross, GA, USA). The samples were subjected to a pre-treatment for degassing of 200 °C for 2 h. Sample composition was determined on an X-ray fluorescence spectrometer (EDX-700, Shimadzu, Barueri, São Paulo, Brazil).

Thermal analysis was performed at a temperature range of 24 to 1000 °C in a platinum crucible with a heating rate of 10 °C min^−1^ and gas flow of 50 mL min^−1^ in a SHIMADZU Differential Thermal Analysis (DTA) and Differential Thermogravimetry (DTG) apparatus thermal analyzer (Barueri, São Paulo, Brazil). 

Diffuse reflectance spectrometry was performed on Shimadzu UV-2600 equipment (Barueri, São Paulo, Brazil), and measurements of the band gap reflectance and photon energy spectra were performed on Shimadzu ISR-2600 Plus equipment with integration spheres from the standard of barium sulphate.

### 2.3. Photocatalytic Performance

The synthesized materials were evaluated for the degradation of rhodamine B under ultraviolet radiation (320–280 nm) from a 9-watt mercury lamp. First, 100 mg of photocatalyst was dispersed in a 100 mL solution of dye with an initial concentration of 10 mg L^−1^. The suspension formed was initially kept in the dark for half an hour under agitation and aeration to achieve the adsorption and desorption equilibrium. Aliquots were collected at 30 min intervals and then centrifuged. For these samples, the absorbance was read on a UV-visible spectrophotometer (Thermo Scientific, Evolution array UV-visible spectrophotometer) (Waltham, MA, USA) at a wavelength of 553 nm [26].

## 3. Results and Discussion

### 3.1. X-Ray Diffraction (XRD)

**Figure 1:**
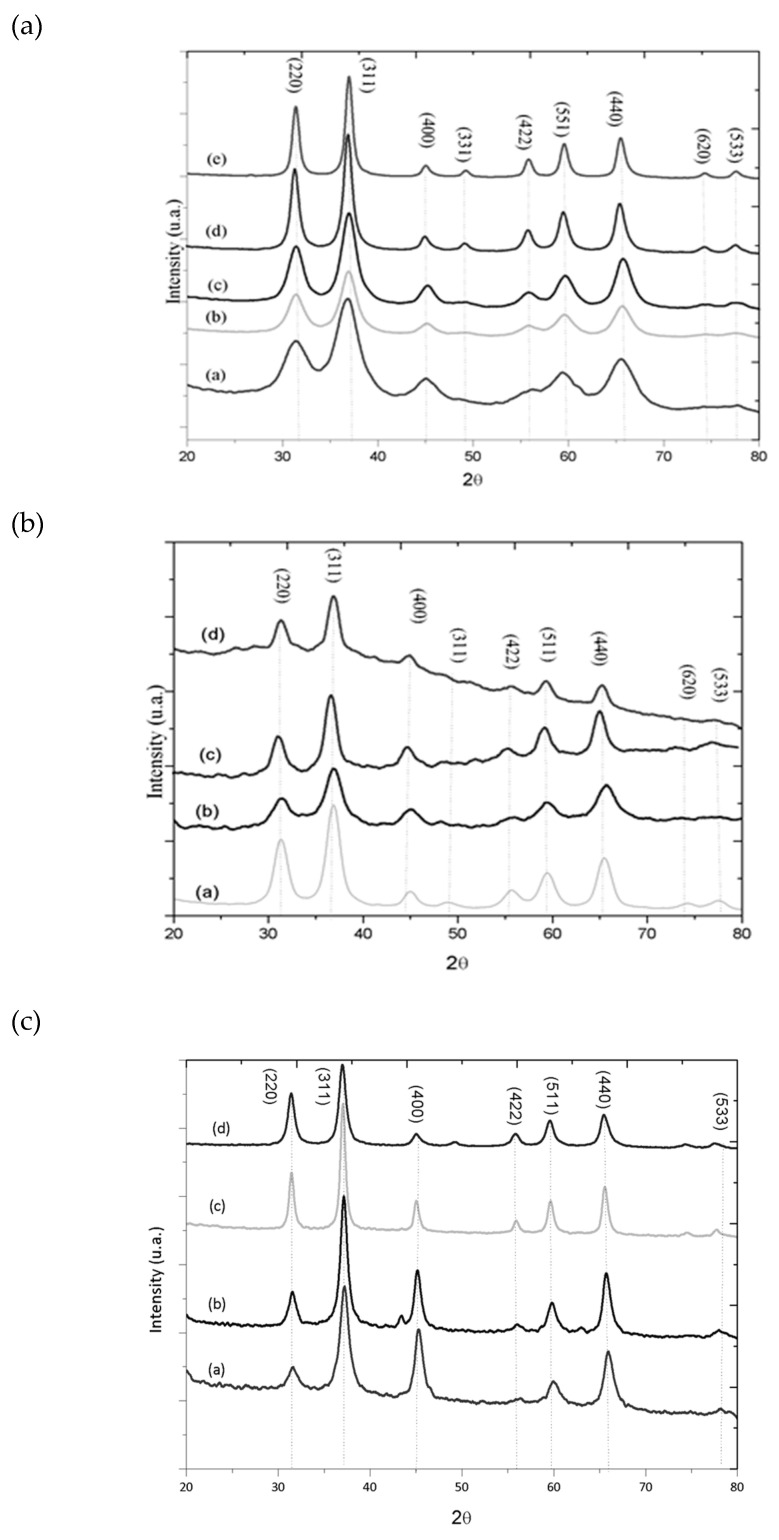
X-ray diffractograms of the samples obtained using the metal-chitosan complexation method: (**a**)—(a) ZnAl_2_O_4_–550, (b) ZnAl_2_O_4_–650, (c) ZnAl_2_O_4_–750, (d) ZnAl_2_O_4_–850, (e) ZnAl_2_O_4_–950; (**b**)—(a) Co_0.1_Zn_0.9_Al_2_O_4_–750, (b) Co_0.5_Zn_0.5_Al_2_O_4_–750, (c) Co_0.1_Zn_0.9_Al_2_O_4_–750, (d) CoZnAl_2_O_4_–750; (**c**)—(a) NiAl_2_O_4_–950, (b) Ni_0.9_Zn_0.1_Al_2_O_4–_950, (c) Ni_0.5_Zn_0,5_Al_2_O_4_–950, (d) Ni_0.1_Zn_0.9_Al_2_O_4_–950.

The diffractograms for the ZnAl_2_O_4_ samples treated at temperatures from 550 °C to 950 °C are shown in Figure 1. All peaks analyzed could be indexed in the center face cubic structure of ZnAl_2_O_4_ according to JCPDS No. 01-071-0968, which have the following 2θ angles: 31.2°, 36.8°, 44.8°, 49.1°, 55.7°, 59.6°, 65.2°, 74.1° and 77.3°, corresponding to the crystallographic planes (220), (311), (400), (422), (511), (440), (620) and (533), respectively. The diffraction patterns of the prepared materials are also very similar to those presented by Stringhini et al. [25] and Zhu et al. [27]. The increase in the synthesis temperature caused an increase in the crystallinity of the samples, characterized by an increase in peak intensity and narrowing of the width by half height, which may be associated with the average size of the crystallite.

The diffractograms for the cobalt-substituted zinc aluminate samples Co_x_Zn_1−x_Al_2_O_4_ (x = 0.1, 0.5, 0.9 e 1.0 mol) calcined at 750 °C are shown in Figure 1b; the increase in the substitution of Zn^2+^ ions present in the spinel structure caused a decrease in the intensity of the peaks, indicating a decrease in the crystallinity of the synthesized material, within the employed conditions. When analyzing the network parameter in relation to the degree of substitution of the Zn^2+^ ions in the structure of the zinc aluminate, the lattice parameter increases as the replacement of the ions occurs, since the Co^2+^ ions are larger than the ions Zn^2+^, thus producing larger particles [5,12,13]. All studied x-values were indexed according to JCPDS N 01-082-2252 for CoAl_2_O_4_. XRD standards for the Ni_x_Zn_1−x_Al_2_O_4_ (x = 0.1, 0.5, 0.9 e 1.0 mol) samples calcined at 950 °C are shown in Figure 1c. For all studied x-values the characteristic spinel phase peaks detected were perfectly indexed according to JCPDS N 01-078-1601 for NiAl_2_O_4_. According to X-ray patterns, no intermediate phase or impurity was observed at any of the proposed x-values; however, at lower temperatures a mixture of NiO and NiAl_2_O_4_ is formed and the spinel phase is not obtained, so this temperature was prioritized for the calcination process of samples replaced by Ni^2+^ ions. However, the increased substitution of zinc ions caused a decrease in peak intensity.

It can be observed that while Zn^2+^ ions were replaced by Ni^2+^ ions, the lattice parameter decreased, ranging from 8.063 to 8.018 Å; similar behavior was described by Han et al. [28]. Porta, Anichini and Bucciarelli [29] observed the same tendency when evaluating the effect of replacing Zn^2+^ ions of zinc aluminate with Ni^2+^ ions, which obtained values ranging from 8.0874 Å, for the pure zinc aluminate sample at 8.0509 Å when the ions were completely replaced.

Kapase et al. [30], Anand et al. [12] and Ianos et al. [31] obtained similar values for the pure ZnAl_2_O_4_ lattice parameter of respectively 8.081, 8.0747 and 8.089 Å. While for the NiAl_2_O_4_ lattice parameter, Dhak and Pramanik [32] obtained 8.0285 Å, Gama et al. [33] obtained from 8.048 Å and Porta, Stone and Turner [34] 8.0514 Å. With this decrease there was a consequent reduction of the unit cell volume from 524 to 516 Å. The lattice parameters and unit cell volumes for all samples are presented in Table 1. For pure zinc aluminate, as the calcination temperature increased, the lattice parameter decreases from 8.124 Å to 8.064 Å.

### 3.2. Diffuse Reflectance Spectrometry

The bandgap of ZnAl_2_O_4_ samples, available in Table 1 (see also Figure S1), showed values of approximately 3.20 eV for unsubstituted samples. The metal-chitosan complexation method produced a material with a smaller energy gap. Therefore, it can be considered that the synthesis method significantly changes the bandgap of the materials.

In the process of replacing Zn^2+^ ions with Ni^2+^, the band interval showed values of 3.12 eV for the fully replaced sample (NiAl_2_O_4_–950) and 3.20 eV for the Ni_0.5_Zn_0.5_Al_2_O_4_–950 sample, showing no major changes compared to the unsubstituted sample (ZnAl_2_O_4_–950). The decrease in the bandgap value is associated with the amount of ions that were replaced, in the proportion of 0.1:0.9, with ions in the amount of 0.1 acting as doping of ZnAl_2_O_4_ by the ions Ni^2+^ and Co^2+^. The difference in the dopant’s electronic structure causes a decrease in the bandgap value [12]. The same effect occurs in the replacement of nickel ions in NiAl_2_O_4_. In samples replaced by cobalt ions, the bandgap value increased as the amount of cobalt ions increases. For Co_0.1_Zn_0.9_Al_2_O_4_–750, the bandgap varied from 2.19 to 3.90 eV when the Zn^2+^ ions were completely replaced; similar values of bandgap were reported by Anand, Kennedy and Vijaya [12].

### 3.3. Infrared Absorption Spectroscopy

**Figure 2:**
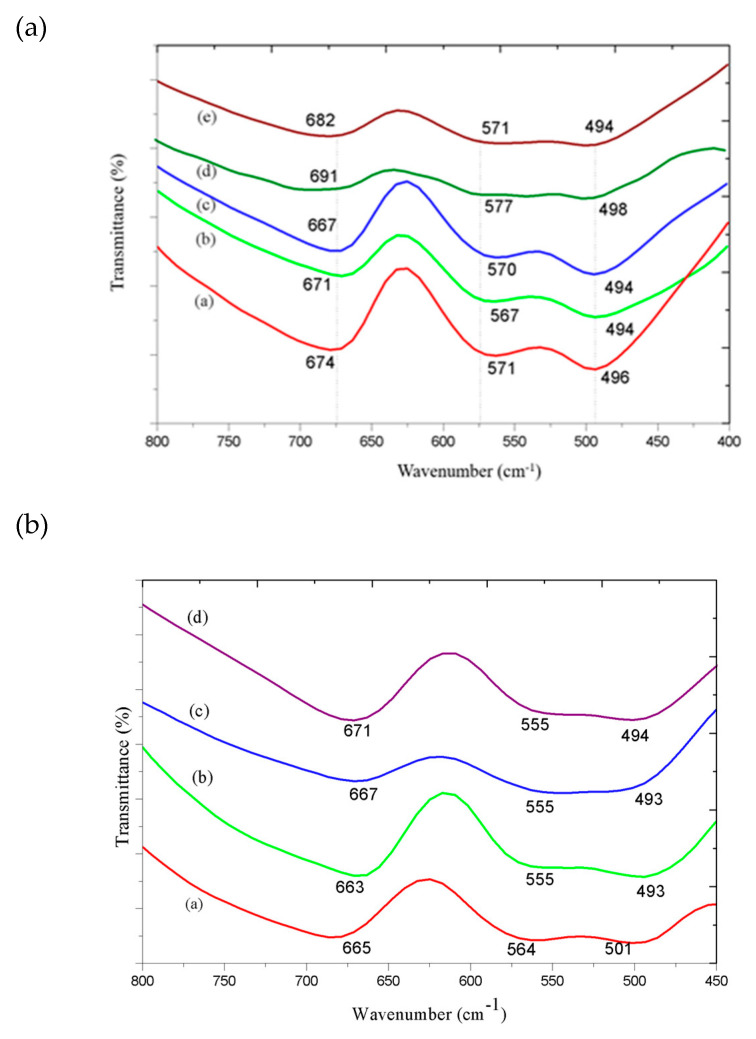

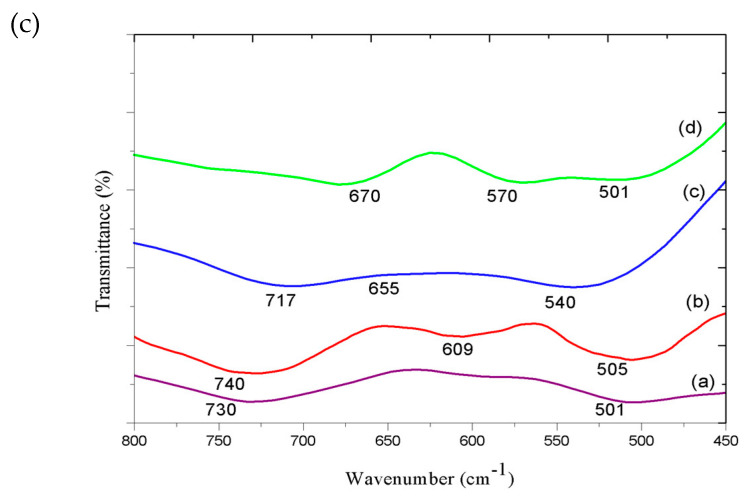
Infrared with Fourier transform of the samples obtained uisng the metal-chitosan complexation method: (**a**)—(a) ZnAl_2_O_4_–550, (b) ZnAl_2_O_4_–650, (c) ZnAl_2_O_4_–750, (d) ZnAl_2_O_4_–850, (e) ZnAl_2_O_4_–950; (**b**)—(a) CoAl_2_O_4_–750, (b) Co_0.9_Zn_0.1_Al_2_O_4_–750, (c) Co_0.5_Zn_0.5_Al_2_O_4_–750, (d) Co_0.1_Zn_0.9_Al_2_O_4_–750; (**c**)—(a) NiAl_2_O_4_–950, (b) Ni_0.9_Zn_0.1_Al_2_O_4_–950, (c) Ni_0.5_Zn_0.5_Al_2_O_4_–950, (d) Ni_0.1_Zn_0.9_Al_2_O_4_–950.

Figure 2 shows the infrared spectrum with Fourier transform (FT-IR) for the synthesized samples. Spinel is characterized by having three bands in the 800 to 450 cm^−1^ range, which confirms the formation of the structure, as these bands are typical of interactions between Al-O at octahedral sites and Zn-O at tetrahedral sites [12,25,35]. The bands located in the 400 to 500 cm^−1^ range are characteristic of the vibrations referring to the interactions between metals and oxygen occupying octahedral sites, and the bands in the 550 to 700 cm^−1^ range are characteristic bands of the vibrations between the metal and oxygen occupying tetrahedral sites [15]. In Figure 2a, vibrations close to ~494 cm^−1^ can be attributed to the vibrations of the group [Al^3+^O_6_] occupying the octahedral sites, and the bands ~680 cm^−1^ and ~570 cm^−1^ are typical of Zn-O connections occupying the tetrahedral sites. The formed material presents a normal spinel structure, due to the absence of vibrations ~730 cm^−1^ referring to interaction (Al^3+^O_4_) at the tetragonal sites [6,12].

The spectra of the samples replaced by Co^2+^ ions, Figure 2b, presented the vibrational modes in about ~670, ~560 and ~494 cm^−1^. The vibrations at ~494 cm^−1^ are attributed to the vibrations of Al^3+^ ions occupying octahedral sites [Al^3+^O_6_], while the bands at ~670 and ~560 cm^−1^ are attributed to the tetrahedral sites being occupied by the Zn^2+^ and Co^2+^ ions (Zn^2+^O_4_:Co^2+^O_4_), confirming the formation of the spinel phase for the replaced samples, being classified as normal spinel [11,31].

The spectra of the samples replaced by Ni^2+^ ions, Figure 2c, showed vibrational modes characteristic of the spinel phase in the region of ~497, 560 and 650 cm^−1^, thus confirming the formation of the normal spinel phase for the sample Ni_0.1_Zn_0.9_Al_2_O_4_–950; however, the Zn_1-x_Ni_x_Al_2_O_4_ samples (x = 0.5, 0.9 and 1.0 mol) showed a vibration around ~730 cm^−1^ characteristic for Ni^2+^ occupying the octahedral site, and the replacement of Zn^2+^ ions by Ni^2+^ ions produced an inverted spinel.

### 3.4. Energy Dispersion Spectroscopy (EDS)

The chemical composition of the samples was determined using energy dispersion spectroscopy (EDS). Table 2 shows the percentages of the metal oxides obtained. Based on these data it can be concluded that the synthesized material has a high degree of purity, with an impurity range for pure ZnAl_2_O_4_ of from 0.08% to 0.26%.

Co_0.1_Zn_0.9_Al_2_O_4_–750 and Co_0.5_Zn_0.5_Al_2_O_4_–750 showed 0.26% and 0.11% of impurities, respectively. The sample with x = 0.9 of cobalt ions was completely pure. For ZnAl_2_O_4_ replaced by 0.1 of Ni^2+^ ions, it was quantified with a total of 0.65%, while nickel ion samples 0.5 and 0.9 presented impurity of 0.15% and 0.14%, respectively. The obtained molar stoichiometry is consistent with the proposed theoretical values.

### 3.5. Scanning Electron Microscopy (SEM) and Transmission Electron Microscopy (TEM)

**Figure 3:**
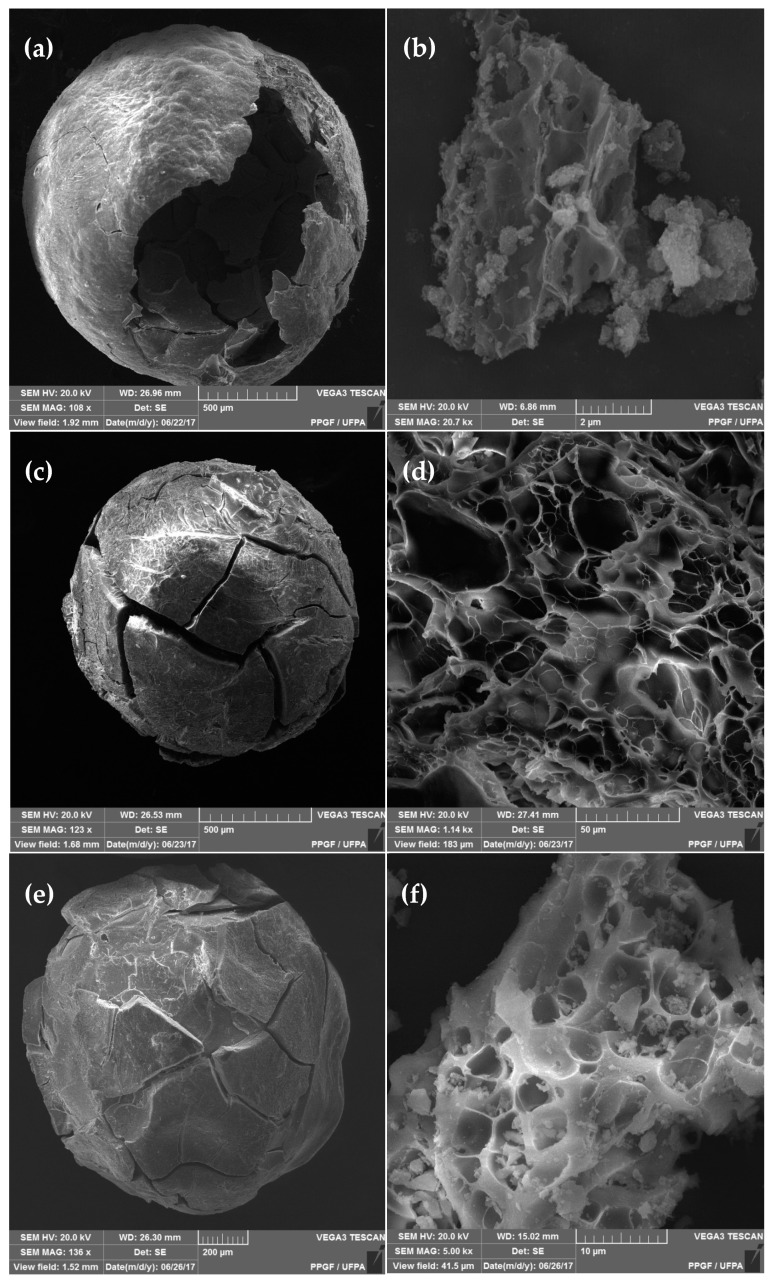
Scanning electron micrographs of samples (**a**) and (**b**) ZnAl_2_O_4_–750; (**c**) and (**d**) NiAl_2_O_4_–950; (**e**) and (**f**) CoAl_2_O_4_–750.

All samples produced were spherical in shape before (Figure S2) and after calcination. Samples replaced by Zn^2+^ and Co^2+^ ions exhibited a similar profile to those of pure aluminates. Therefore, SEM analyses were performed for ZnAl_2_O_4_, NiAl_2_O_4_ and CoAl_2_O_4_ samples. According to the analyses, it can be concluded that the spheres formed after calcination are hollow and exhibit cracks on the surface, justifying the synthesis process based on chitosan as a template, and its elimination during calcination produces a highly porous spherical structure with low mechanical resistance. The materials have a sponge-like porosity, as shown in Figure 3b,d,f.

**Figure 4:**
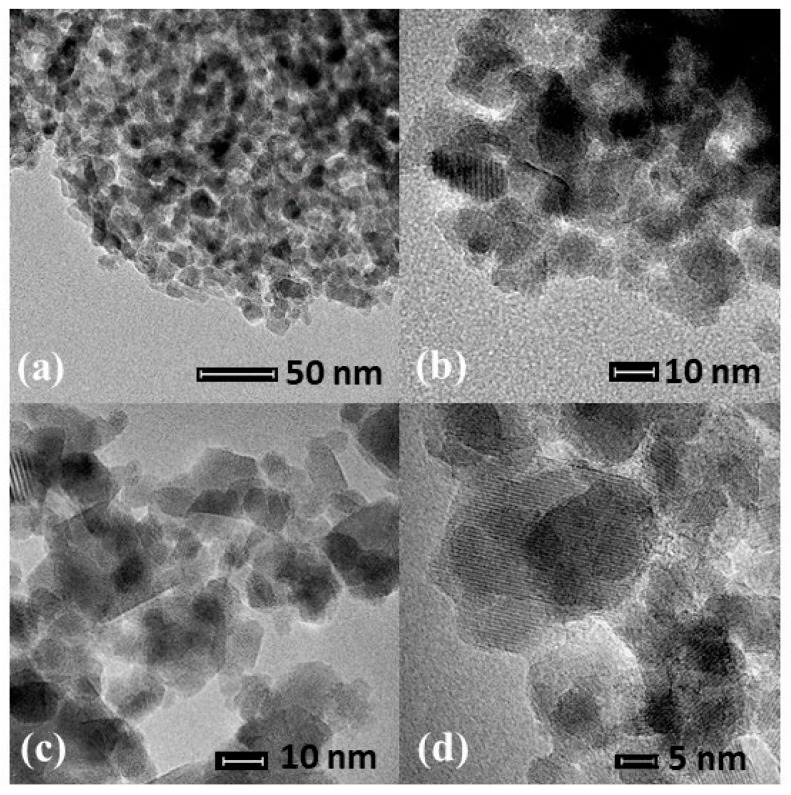
Transmission electron microscopy (TEM) of samples (**a**) ZnAl_2_O_4_–750; (**b**) NiAl_2_O_4_–950; (**c**) CoAl_2_O_4_–750; (**d**) NiAl_2_O_4_–950.

Figure 4 shows the transmission micrographs for the ZnAl_2_O_4_–750, NiAl_2_O_4_–950 and CoAl_2_O_4_–750 samples. The images show that the zinc, cobalt and nickel aluminate particles are uniform and nanoaggregated; The zinc metal ions using Co^2+^ and Ni^2+^ showed no significant changes in material morphology.

### 3.6. N_2_ Adsorption-Desorption Isotherms

**Figure 5:**
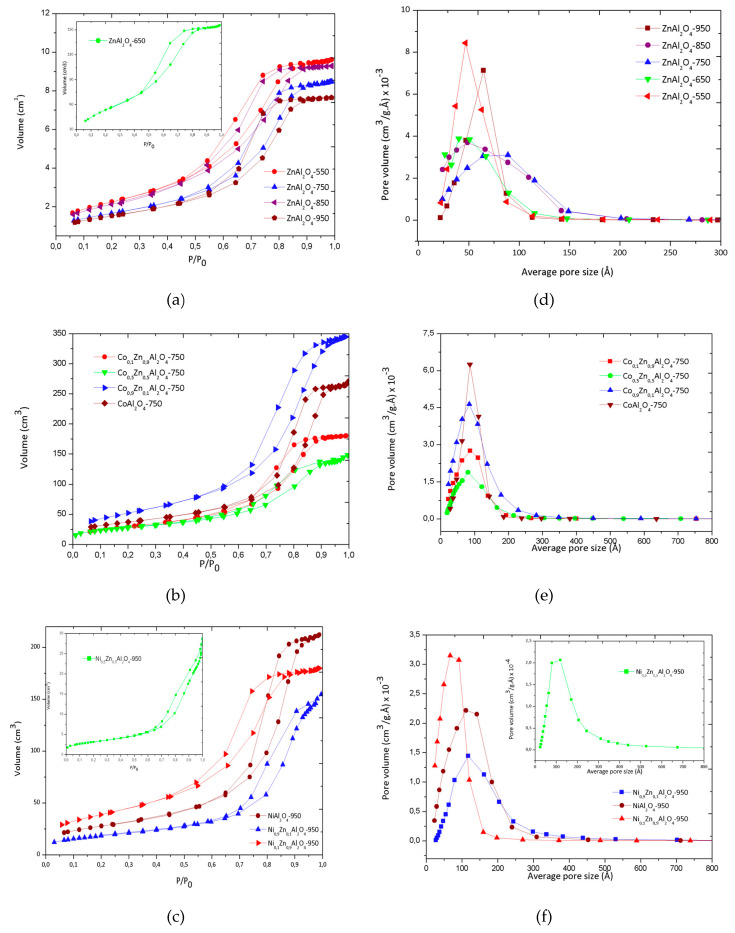
N_2_ adsorption-desorption isotherms and average pore size distribution. N_2_ adsorption-desorption isotherms for: (**a**) ZnAl_2_O_4_ samples, (**b**) samples replaced by cobalt ions. (**c**) samples replaced by nickel ions. Average pore size distribution for: (**d**) ZnAl_2_O_4_ samples, (**e**) samples replaced by cobalt ions, (**f**) samples replaced by nickel ions.

Adsorption-desorption isotherms of N_2_, Figure 5a–c, showed a similar profile. According to the IUPAC classification, these isotherms can be defined as type IV isotherms because they have a characteristic hysteresis cycle of mesoporous structure [36]. The mesoporosity of the samples was confirmed by analyzing the average pore size distribution using the Barrett-Joyner-Halenda (BJH) method. Average pore size distribution graphs are available in Figure 5d–f, which show a predominant distribution in the mesoporous structure region (2 < average pore size < 50 nm) [22].

Figure 5d shows the average pore size distribution for the zinc aluminate samples, synthesized at a calcination temperature of 550 to 950 °C. It can be seen that they all had a unimodal distribution, with ZnAl_2_O_4_–550 and ZnAl_2_O_4_–950 having a narrow unimodal with pore size ranging close to 4.5 nm and 6.5 nm, respectively. The distribution is similar to that obtained by Anchieta and collaborators [15]. The samples ZnAl_2_O_4_–650, ZnAl_2_O_4_–750 and ZnAl_2_O_4_–850 showed, however, a wide unimodal distribution, with mean pore values of 4 to 5 nm, 6.5 to 9 nm and 5 to 7 nm, respectively.

Figure 5b shows the N_2_ adsorption-desorption isotherms for cobalt substituted samples at the theoretical molar ratio of 0.1, 0.5, 0.9 and 1.0; they all presented type IV isotherms similar to those obtained for unsubstituted ZnAl_2_O_4_ samples, which was confirmed by the analysis of the average pore size.

The replacement of Zn^2+^ ions in the structure of zinc aluminate caused changes in its textural properties. By replacing 0.1 of Zn^2+^ ions with Co^2+^ ions the surface area decreases from 175 to 109 m^2^ g^−1^. Similarly, the total volume of pore size decreased from 0.331 to 0.282 cm^3^ g^−1^; however, the average pore diameter increased significantly from 59.2 to 74.4 Å. According to the data obtained by the BET/BJH analysis, the increase in the calcination temperature linearly caused a decrease in the surface area of the ZnAl_2_O_4_ samples.

Replacing zinc ions with cobalt metal ions increased the surface area of the material to 193 m^2^ g^−1^ for the sample replaced with 0.9 of Co^2+^ ions, but the CoAl_2_O_4_–750 sample had an intermediate surface area of 135 m^2^ g^−1^ and an average pore size of 110 Å. Table 3 presents the data regarding surface area, average pore diameter and total pore volume for all materials obtained.

Replacing 0.1 of the Zn^2+^ ions in the ZnAl_2_O_4_ structure with Ni^2+^ ions in the Ni_0.1_Zn_0.9_Al_2_O_4_–750 sample caused a significant change in their properties. The surface area decreases from 175 m^2^ g^−1^ in the ZnAl_2_O_4_–750 sample to 141 m^2^ g^−1^, while with the replacement the mean pore diameter increased from 59.2 Å to 78.3 Å. However, the total pore volume decreased from 0.331 cm^3^ g^−1^ to 0.285 cm^3^ g^−1^.

The results obtained using BET/BJH demonstrate that the metal-chitosan complexation synthesis method is highly efficient in obtaining materials with a porous structure in comparison with other methods used for the synthesis of aluminates. Wei and Chen [37], using the sol-gel method, obtained ZnAl_2_O_4_ with a surface area of 58 m^2^ g^−1^ and pore volume of 0.0029 cm^3^ g^−1^. Queiroz et al. [35] synthesized ZnAl_2_O_4_ using the polymeric precursor method and obtained a very low surface area ranging from 8 to 77 m^2^ g^−1^ and a total pore volume ranging from 0.004 to 0.027 cm^3^ g^−1^, respectively.

However, in the research carried out by Nuernberg et al. [21], MgAl_2_O_4_ synthesized using metal-chitosan complexation presented a surface area of 168 m^2^ g^−1^ and 0.311 cm^3^ g^−1^ of total pore volume, and such results contributed to better catalytic performance in the direct decomposition of methane. According to Anchieta et al. [15] high surface area and pore size are essential characteristics for catalytic purposes. In their research they obtained zinc aluminate with 158 m^2^ g^−1^ and 0.302 cm^3^ g^−1^ for surface area and total pore volume, respectively.

Faletto et al. [18] further demonstrated that ZnAl_2_O_4_ synthesized using different methods (co-precipitation, hydrothermal and micro-hydrothermal) alters the structural properties and that the pore size of the catalyst particles strongly influences the efficiency of the photocatalytic process in the degradation of textile dyes, like reactive red 141. Their results indicated that high photocatalytic activity was obtained for materials with larger pore sizes.

Thus, in this work, we note that the use of the metal-chitosan complexation method produced catalysts with larger pore size and volume and high surface area, as shown by the BET/BJH results. Such characteristics can probably contribute to a better photocatalytic performance.

### 3.7. Thermogravimetric Analysis (TG) and Differential Thermal Analysis (DTA)

**Figure 6:**
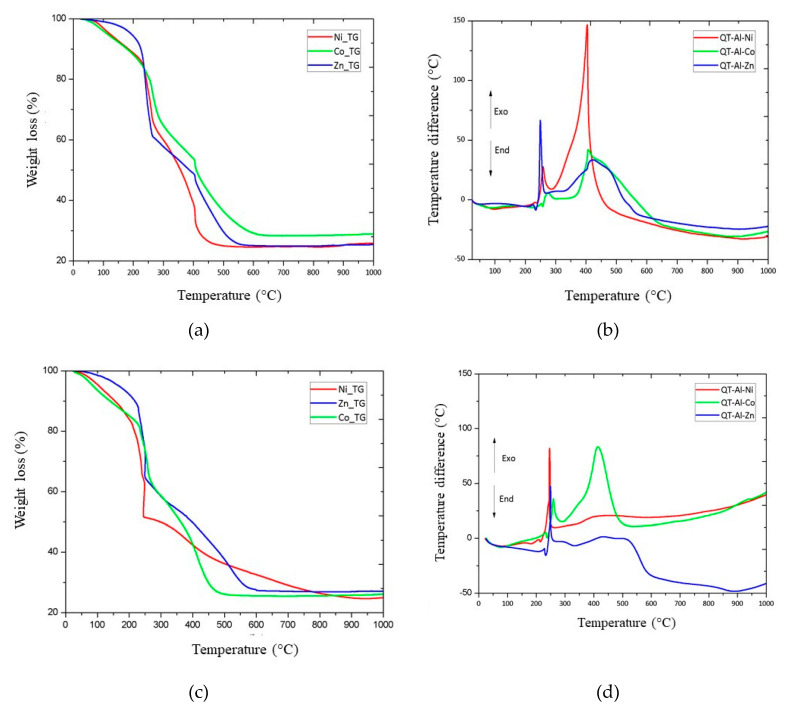
Thermogravimetric analysis of the samples QT-Al-Zn, QT-Al-Ni and QT-Al-Co under (**a**) air and (**c**) nitrogen. Differential thermal analysis for QT-Al-Zn, QT-Al-Ni and QT-Al-Co samples under (**b**) air and (**d**) nitrogen. Obtained in the temperature range of approximately 24 to 1000 °C, with a heating rate of 10 °C min^−1^ and synthetic air flow of 50 mL min^−1^.

The chitosan-aluminum-cobalt (QT-Al-Co) and chitosan-aluminum-nickel (QT-Al-Ni) samples submitted to thermogravimetric analysis under synthetic air and nitrogen gas atmosphere, available in Figure 6, presented three distinct events of weight loss (Figures S3 and S4). For the samples submitted to synthetic air, the first event occurred in the temperature range of 75 to 114 °C for the cobalt containing sample and from 74 to 105 °C for the nickel complex. This event can be attributed to the loss of adsorbed water and the elimination of physically adsorbed gases from the sample surface.

For the chitosan-aluminum-cobalt complex this loss was 7.0%, for the chitosan-aluminum-nickel complex this loss reached 11.4%. However, the sample formed by the chitosan-aluminum-zinc complex showed no losses at this temperature [11,26].

By switching the purge gas to nitrogen, the TG curves showed a similar degradation profile. QT-Al-Co and QT-Al-Ni samples showed three events. QT-Al-Co presented the first event regarding the elimination of adsorbed water in the range of 71 to 105 °C with a weight loss of 5.8%. The QT-Al-Ni sample showed 14.4% of weight loss in the range of 44 to 110 °C. The QT-Al-Zn sample did not present this event regarding the loss of water and adsorbed gases.

The second event is related to the thermal deterioration of the nitrate-eliminating carbon chain (NO_x_), which should occur in the range of 200 to 300 °C and is due to partial deterioration of chitosan [38,39]. In QT-Al-Zn, the first event, degradation, began at 213 °C and continued to 357 °C producing a 36.1% weight loss. QT-Al-Ni showed a weight loss of 27.9%, starting at a temperature of 238 °C and completing at 263 °C. The QT-Al-Co sample presented a 34.8% weight loss from 239 to 352 °C, when submitted to synthetic air atmosphere. When using nitrogen gas, QT-Al-Zn presented the first event with a weight loss of 43.1% in the range of 243 to 250 °C. QT-Al-Co showed thermal degradation of 42.9% in the temperature range of 215 to 261 °C, while the QT-Al-Ni sample had a weight loss of 32.3% in the temperature range of 230 to 260 °C.

The third event was attributed to the total burning of charred waste that normally occurs between 400 and 600 °C [40,41]. For this event, the start and end temperatures were 371 and 414 °C, 379 and 506 °C and 213 and 561 °C for QT-Al-Ni, QT-Al-Co and QT-Al-Zn, respectively. The weight loss was 29.6% for QT-Al-Co, 38.5% for QT-Al-Ni and 35.7% for QT-Al-Zn. For the QT-Al-Ni, QT-Al-Co and QT-Al-Zn samples, submitted to nitrogen gas atmosphere, they presented a weight loss of 26.9%, 25.8% and 29.4% respectively. This event occurred in the range of 386 to 549 °C for the nickel-containing sample, for QT-Al-Co samples it was processed in the range of 325 to 596 °C and for the QT-Al-Zn samples it occurred in the range of 315 to 600 °C.

### 3.8. Photocatalytic Performance

**Figure 7:**
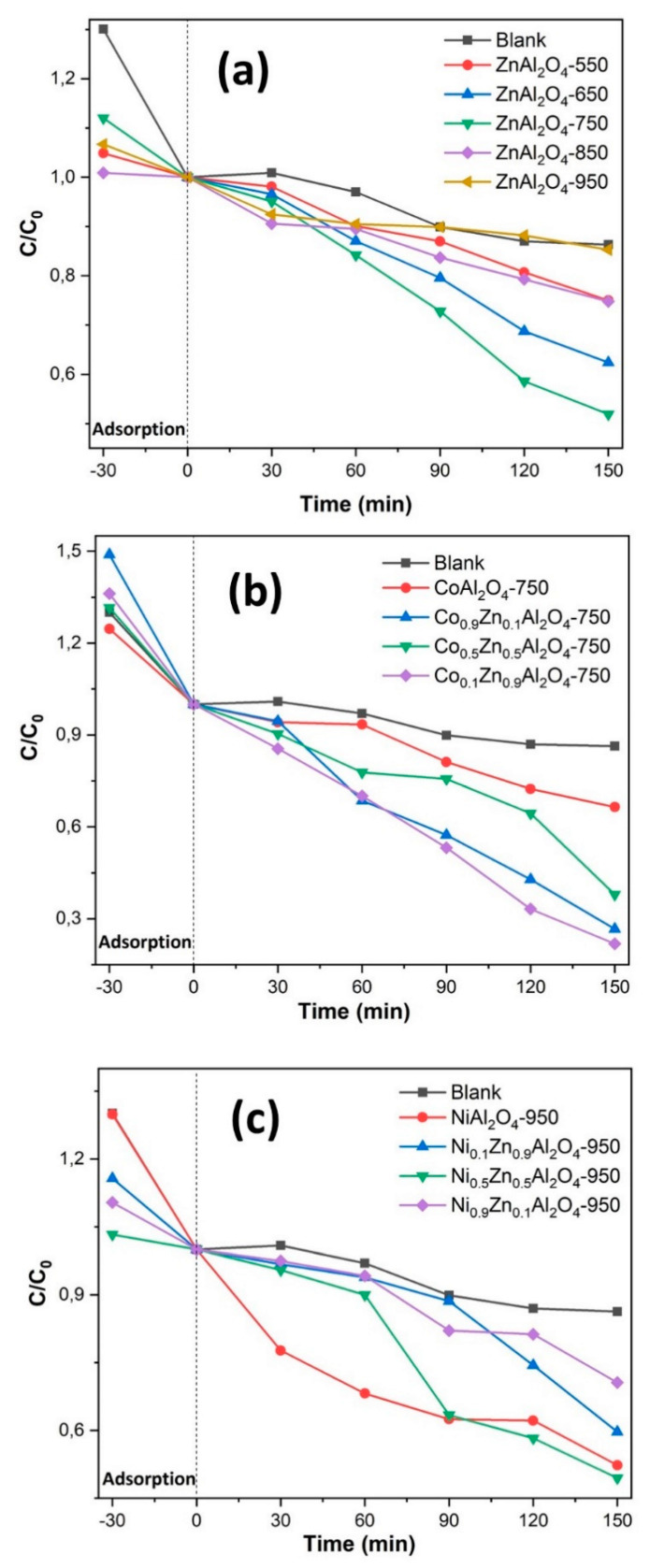
Rhodamine B photocatalytic degradation test for samples: ZnAl_2_O_4_–550, ZnAl_2_O_4_–650, ZnAl_2_O_4_–750, ZnAl_2_O_4_–850, ZnAl_2_O_4_–950 (**a**); CoAl_2_O_4_–750, Co_0.1_Zn_0.9_Al_2_O_4_–750, Co_0.5_Zn_0.5_Al_2_O_4_–750, Co_0.9_Zn_0.1_Al_2_O_4_–750 (**b**); NiAl_2_O_4_–950, Ni_0.1_Zn_0.9_Al_2_O_4_–950, Ni_0.5_Zn_0.5_Al_2_O_4_–950, Ni_0.9_Zn_0.1_Al_2_O_4_–950 (**c**) and blank (direct photolysis).

According to Figure 7, a difference in the dye degradation rate can be observed as the calcination temperature of the ZnAl_2_O_4_ samples changed. Samples ZnAl_2_O_4_–550, ZnAl_2_O_4_–650, ZnAl_2_O_4_–750, ZnAl_2_O_4_–850 and ZnAl_2_O_4_–950, Figure 7a, had a removal rate of 59%, 56%, 48%, 47% and 15%, respectively, at a reaction time of 150 min. The greatest separation between degradation rates occurred within 90 min from the beginning of the photocatalytic test, where a degradation of about 27%, 37%, 53%, 38% and 6% of the dye was observed for calcined zinc aluminate at temperatures of 550, 650, 750, 850 and 950 °C, respectively.

For the Ni_0.1_Zn_0.9_Al_2_O_4_–950, Ni_0.5_Zn_0.5_Al_2_O_4_–950, Ni_0.9_Zn_0.1_Al_2_O_4_–950 and NiAl_2_O_4_–950 samples, presented in Figure 7c, the samples Ni_0.1_Zn_0.9_Al_2_O_4_–950, Ni_0.5_Zn_0.5_Al_2_O_4_–950, Ni_0.9_Zn_0.1_Al_2_O_4_–950 and NiAl_2_O_4_–950 showed a removal rate of 64%, 51%, 55% and 66%.

The zinc aluminate samples replaced by Co^2+^ ions, presented in Figure 7b, showed a 83% removal for Co_0.1_Zn_0.9_Al_2_O_4_–750, 63% for Co_0.5_Zn_0.5_Al_2_O_4_–750 and 73% for Co_0.9_Zn_0.1_Al_2_O_4_–750, and the CoAl_2_O_4_–750 sample presented a degradation rate of 35% of rhodamine B dye. The highest degradation rate between the dyes was observed at 120 min reaction time with an 83%, 36%, 57% and 23% rate for samples replaced with 0.1, 0.5, 0.9 and 1.0 mol cobalt, respectively. Table 4 presents the rhodamine B dye removal rates for all photocatalysts evaluated.

The increase in the removal rate of zinc aluminate substituted by 0.1 cobalt ions may be justified by the decrease of bandgap energy, material, and its high surface area, in relation to unsubstituted samples. However, the nickel ion-replaced samples did not show a significant increase in the degradation rate, and this may be attributed to the fact that the substitution did not cause significant changes in bandgap, but also the fact that the high synthesis temperature reduced the surface area of the samples. The degradation rate for rhodamine B dye using photolysis were 13% after 150 min under ultraviolet radiation exposure. Ragul and Sumathi [42] reported a degradation rate of 20% using pure zinc aluminate as a photocatalyst when degrading rhodamine B dye.

In the photodegradation process, the photocatalyst promotes a variety of photoinduced chemical reactions that occur in the presence of O_2_ and H_2_O, and the products formed are capable of promoting the degradation of organic pollutants [43]. In the literature, the ZnAl_2_O_4_ bandgap is theoretically reported to be 3.8 eV; however, some studies report a bandgap greater than 5.0 eV. Therefore, ultraviolet light becomes necessary to activate the photocatalyst [3,43].

The following diagram shows the events that can occur on the surface of Zn_1−x_M_x_Al_2_O_4_ (M = Co^2+^, Ni^2+^) until photodegradation of rhodamine B [44,45,46]. When light is absorbed by Zn_1−x_M_x_Al_2_O_4_ (M = Co^2+^, Ni^2+^) two charge carriers are generated, electrons (e^−^) and positive holes (h^+^).

(1) $$Zn_{1-x}M_{x}Al_{2}O_{4}+hv\to Zn_{1-x}M_{x}Al_{2}O_{4}\left(h^{+}\right)+Zn_{1-x}M_{x}Al_{2}O_{4}\left(e^{-}\right)$$

The photogenerated holes can react with water (Equation (2)) and with hydroxide ions that are adsorbed on the surface of the material producing hydroxyl radical (Equation (3)).

(2) $$Zn_{1-x}M_{x}Al_{2}O_{4}\left(h^{+}\right)+H_{2}O\to OH^{•}+H^{+}+Zn_{1-x}M_{x}Al_{2}O_{4}$$

(3) $$Zn_{1-x}M_{x}Al_{2}O_{4}\left(h^{+}\right)+HO^{-}\to OH^{•}+Zn_{1-x}M_{x}Al_{2}O_{4}$$

The electrons formed in the process of excitation with radiation react with the dissolved molecular oxygen forming superoxide anions (Equation (4)) followed by the protonation of the superoxide (Equation (5)).

(4) $$Zn_{1-x}M_{x}Al_{2}O_{4}\left(e^{-}\right)+O_{2}\to {O_{2}}^{•}+Zn_{1-x}M_{x}Al_{2}O_{4}$$

(5) $$O_{2}^{•-}+H^{+}\to HOO^{•}$$

When the photocatalytic decomposition process is complete, the photo-generated radicals promote the complete decomposition of the organic pollutant in carbon dioxide and water (Equation (6)).

(6) $$OH^{•}+{O_{2}}^{•}+HOO^{•}+Rhodamine\;B\to CO_{2}+H_{2}O$$

### 3.9. Kinetic Degradation Studies

**Figure 8:**
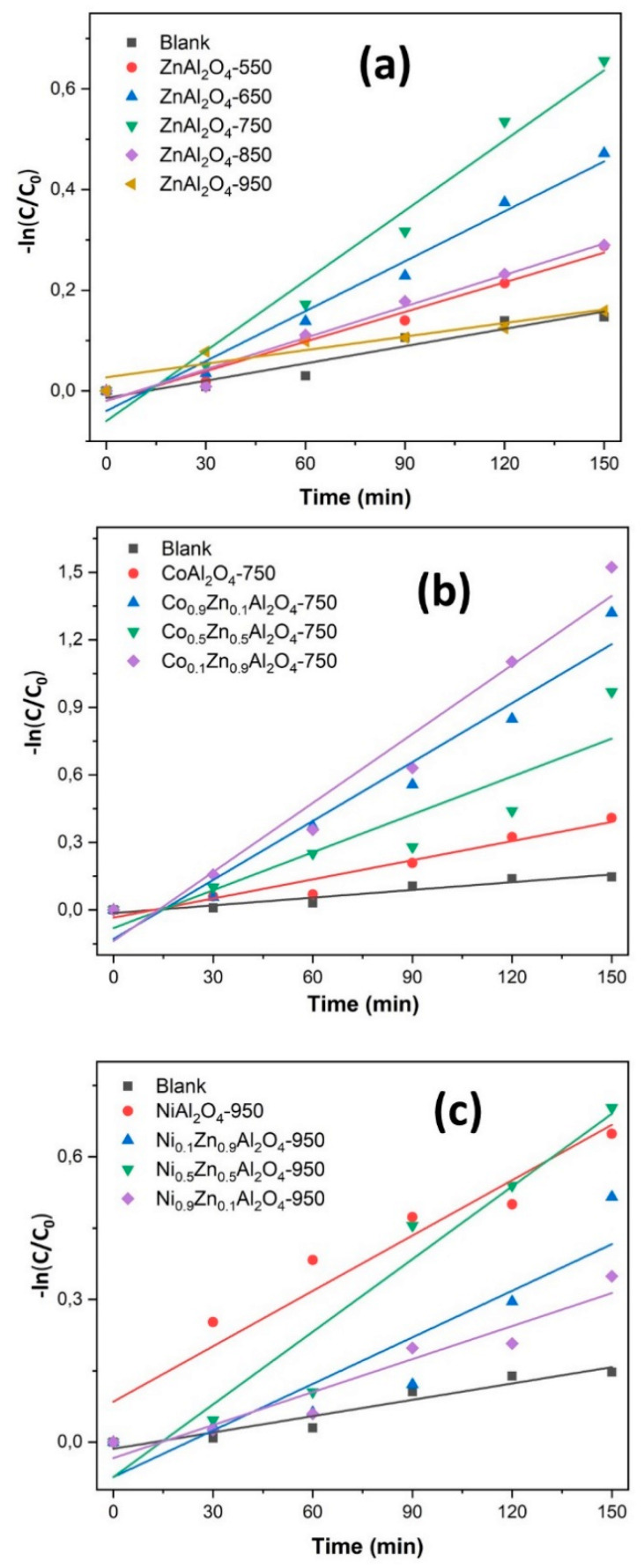
Rhodamine B photocatalytic degradation kinetics for samples: ZnAl_2_O_4_–550, ZnAl_2_O_4_–650, ZnAl_2_O_4_–750, ZnAl_2_O_4_–850, ZnAl_2_O_4_–950 (**a**); CoAl_2_O_4_–750, Co_0.1_Zn_0.9_Al_2_O_4_–750, Co_0.5_Zn_0.5_Al_2_O_4_–750, Co_0.9_Zn_0.1_Al_2_O_4_–750 (**b**); NiAl_2_O_4_–950, Ni_0.1_Zn_0.9_Al_2_O_4_–950, Ni_0.5_Zn_0.5_Al_2_O_4_–950, Ni_0.9_Zn_0.1_Al_2_O_4_–950 (**c**) and Blank (direct photolysis).

The photodegradation reactions of organic pollutants follow first-order degradation kinetics. In this case, it is suggested that the degradation rate following pseudo-first-order kinetics can be explained using a Langmuir-Hinshelwood model [15,47]. As seen in Figure 8, the relationship between −ln (C/C_0_) demonstrates that the degradation of rhodamine B catalyzed by most of the synthesized photocatalysts agrees with pseudo-first-order kinetics.

The photodegradation rate (r) of rhodamine B after the adsorption equilibrium can be expressed by Equation (7):

(7) $$ln\left(C/C_{0}\right)=-kt$$

where C_0_ is the initial concentration of the solution after reaching the adsorption equilibrium, C is the concentration at time t, and k is the reaction rate constant for the photodegradation process [7,48].

Table 4 presents the values of the degradation rate constant (k) and the rhodamine B dye removal rate. With these values, it can be concluded that the dye photodegradation process for most of the photocatalysts in question follows pseudo-first-order kinetics.

## 4. Conclusions

Zn_1-x_M_x_Al_2_O_4_ was successfully obtained using the metal-chitosan complexation method and Zn^2+^ ions were gradually replaced by Co^2+^ and Ni^2+^. XRD and FTIR analysis confirmed the formation of the spinel phase and the high degree of purity in all synthesized samples. Mesoporous Zn_1-x_M_x_Al_2_O_4_ was developed with a smaller band interval and a greater number of reaction sites available for rhodamine B photodegradation. Here, we investigated for the first time the photocatalytic activity of Zn_1-x_M_x_Al_2_O_4_ obtained using metal-chitosan complexation method and the effect of replacing Zn^2+^ ions with Ni^2+^ and Co^2+^ on the degradation of rhodamine B. The photocatalytic activity was investigated under ultraviolet radiation. ZnAl_2_O_4_–950 and Co_0.1_Zn_0.9_Al_2_O_4_–750 samples had the lowest (15%) and highest (83%) dye degradation rates, respectively. Rhodamine B photodegradation followed pseudo-first-order kinetics.

## Figures and Tables

**Table 1:** Bandgap, lattice parameter and unit cell volume for samples Zn_1−x_M_x_Al_2_O_4_.

Sample	Bandgap (eV)	Lattice Parameter, a (Å)	Unit Cell Volume, a^3^, (Å^3^)
ZnAl_2_O_4_–550	3.30	8.124	536
ZnAl_2_O_4_–650	3.04	8.080	528
ZnAl_2_O_4_–750	3.16	8.070	526
ZnAl_2_O_4_–850	3.27	8.066	525
ZnAl_2_O_4_–950	3.19	8.064	524
Ni_0.1_Zn_0.9_Al_2_O_4_–950	2.98	8.060	524
Ni_0.5_Zn_0.5_Al_2_O_4_–950	3.20	8.034	519
Ni_0.9_Zn_0.1_Al_2_O_4_–950	2.78	8.031	518
NiAl_2_O_4_–950	3.12	8.018	516
Co_0.1_Zn_0.9_Al_2_O_4_–750	2.19	8.074	526
Co_0.5_Zn_0.5_Al_2_O_4_–750	2.21	8.088	529
Co_0.9_Zn_0.1_Al_2_O_4_–750	2.51	8.091	530
CoAl_2_O_4_–750	3.90	8.103	532

**Table 2:** X-ray fluorescence spectroscopy quantification and energy dispersion spectroscopy.

Sample Theoretical	EDX Al_2_O_3_ (%)	EDX Co_2_O_3_ (%)	EDX ZnO (%)	EDX NiO (%)	EDX Fe_2_O_3_ (%)	EDX CaO (%)
ZnAl_2_O_4_–550	65.8	—	23.7	—	0.08	—
ZnAl_2_O_4_–650	59.9	—	28.5	—	0.08	0.26
ZnAl_2_O_4_–750	63.9	—	24.1	—	0.06	0.11
ZnAl_2_O_4_–850	70.7	—	27.2	—	0.14	—
ZnAl_2_O_4_–950	74.0	—	21.8	—	—	—
CoAl_2_O_4_–750	62.2	32.4	—	—	—	—
NiAl_2_O_4_–950	71.3	—	—	25.3	—	—
Co_0.1_Zn_0.9_Al_2_O_4_–750	58.6	5.1	31.4	—	0.12	0.14
Co_0.5_Zn_0.5_Al_2_O_4_–750	50.6	41.3	15.1	—	0.11	—
Co_0.9_Zn_0.1_Al_2_O_4_–750	52.3	41.3	1.9	—	—	—
Ni_0.1_Zn_0.9_Al_2_O_4_–950	60.5	—	29.8	2.8	0.30	0.35
Ni_0.5_Zn_0.5_Al_2_O_4_–950	69.1	—	18.8	11.5	0.15	—
Ni_0.9_Zn_0.1_Al_2_O_4_–950	59.2	—	5.8	33.7	0.14	—

**Table 3:** Surface area, average pore diameter and total pore volume of samples Zn_1-x_M_x_Al_2_O_4_.

Sample	Surface Area (m^2^ g^−1^)	Average Pore Size (Å)	Total Pore Volume (cm^3^ g^−1^)
ZnAl_2_O_4_–550	197	45.9	0.255
ZnAl_2_O_4_–650	185	58.3	0.343
ZnAl_2_O_4_–750	175	59.2	0.331
ZnAl_2_O_4_–850	136	69.3	0.300
ZnAl_2_O_4_–950	124	69.3	0.270
NiAl_2_O_4_–950	101	85.4	0.330
Ni_0.9_Zn_0.1_Al_2_O_4_–950	67	129.9	0.234
Ni_0.5_Zn_0.5_Al_2_O_4_–950	30	63.0	0.072
Ni_0.1_Zn_0.9_Al_2_O_4_–950	141	78.3	0.285
CoAl_2_O_4_–750	135	85.2	0.421
Co_0.9_Zn_0.1_Al_2_O_4_–750	193	110.2	0.532
Co_0.5_Zn_0.5_Al_2_O_4_–750	101	82.0	0.227
Co_0.1_Zn_0.9_Al_2_O_4_–750	109	74.4	0.282

**Table 4:** Degradation rate constant (k) and rhodamine B dye removal rate.

Sample	Removal (%)	k (min^−1^) × 10^−3^	R^2^
Blank	13	^—^	^—^
ZnAl_2_O_4_–550	59	2.0	0.944
ZnAl_2_O_4_–650	56	3.3	0.972
ZnAl_2_O_4_–750	48	4.6	0.971
ZnAl_2_O_4_–850	47	1.8	0.946
ZnAl_2_O_4_–950	15	0.9	0.880
NiAl_2_O_4_–750	66	3.8	0.947
CoAl_2_O_4_–950	35	2.5	0.940
Ni_0.1_Zn_0.9_Al_2_O_4_–950	64	3.3	0.855
Ni_0.5_Zn_0.5_Al_2_O_4_–950	51	5.1	0.936
Ni_0.9_Zn_0.1_Al_2_O_4_–950	55	2.3	0.931
Co_0.1_Zn_0.9_Al_2_O_4_–750	83	4.9	0.975
Co_0.5_Zn_0.5_Al_2_O_4_–750	62	4.8	0.957
Co_0.9_Zn_0.1_Al_2_O_4_–750	73	2.9	0.954

## Supplementary Materials

The following are available online at https://www.mdpi.com/1996-1944/13/9/2150/s1, Figure S1. Comparison between UV-Vis diffuse reflectance spectra of samples: ZnAl_2_O_4_–550, ZnAl_2_O_4_–650, ZnAl_2_O_4_–750, ZnAl_2_O_4_–850, ZnAl_2_O_4_-950, Figure S2. Metal spheres complexed with chitosan before calcination: (a) QT-Zn-Al, (b) QT-Ni-Al, (c) QT-Co-Al, Figure S3. DTA and TG of pure chitosan in (a) synthetic air and (b) nitrogen under synthetic air atmosphere with a flow of 50 cm^3^ min^−1^ and a heating ratio of 10 °C min^−1^ to a temperature of 1000 °C, Figure S4. TG and DTG curves of pure chitosan in (a) synthetic air and (b) nitrogen gas with a flow of 50 cm^3^/min and heating ratio of 10 °C/min from ~24 °C to 700 °C.
