# Correction: Pontes do Nascimento et al. Synthesis of Mesoporous Zn_1−x_M_x_Al_2_O_4_ Substituted by Co^2+^ and Ni^2+^ Ions and Application in the Photodegradation of Rhodamine B. *Materials* 2020, *13*, 2150

**DOI:** 10.3390/ma17030759

**Published:** 2024-02-05

**Authors:** Nilson Machado Pontes do Nascimento, Bárbara Ronara Machado de Lima, José Roberto Zamian, Carlos Emmerson Ferreira da Costa, Luís Adriano Santos do Nascimento, Rafael Luque, Geraldo Narciso da Rocha Filho

**Affiliations:** 1 Graduation Program in Chemistry, Federal University of Pará, Augusto Corrêa Street, Guamá, Belém 66075-110, PA, Brazil; nilson-pontes@hotmail.com (N.M.P.d.N.); barbararonara@hotmail.com (B.R.M.d.L.); zamian@ufpa.br (J.R.Z.); emmersoncosta@yahoo.com.br (C.E.F.d.C.); adrlui1@yahoo.com.br (L.A.S.d.N.); 2 Laboratory of Catalysis and Oilchemistry, Federal University of Pará, Street Augusto Correia, Guamá, Belém 66075-110, PA, Brazil; 3 Laboratory of Oils of the Amazon, Federal University of Pará, Perimetral Avenue, Guamá, Belém 66075-110, PA, Brazil; 4 Graduation Program in Biotechnology, Federal University of Pará, Augusto Corrêa Street, Guamá, Belém 66075-110, PA, Brazil; 5 Department of Organic Chemistry, Universidad de Córdoba, Ctra Nnal IV-A, Km 396, E14014 Cordoba, Spain

## 1. Error in Figure

In the original publication [1], an error occurred in “**Figure 3**. Scanning electron micrographs of samples (a) and (b) ZnAl_2_O_4_–750; (c) and (d) NiAl_2_O_4_–950; (e) and (f) CoAl_2_O_4_–750”, specifically in Figure 3d. The image from Figure 3b was inadvertently duplicated and used again in Figure 3d. The figure’s label remains unchanged, but the image itself has been replaced. The authors state that the scientific conclusions are unaffected. This correction was approved by the Academic Editor. The original publication has also been updated. Figure 3 should be replaced with the following figure:

## 2. References

In the revised version of this article, two references underwent an exchange. References numbered 4 and 5 required modifications due to changes made during manuscript formatting. These references have been substituted with the following sources, which maintain the same numbering:

- Li, X.; Zhu, Z.; Zhao, Q.; Wang, L. 2011 replaces reference number 4.
- Rahnamaeiyan, S.; Nasiri, M.; Talebi, R.; Khademolhoseini, S. 2015 replaces reference number 5.

Consequently, the reference initially numbered 5 (Kapse, S.D.; Raghuwanshi, F.C.; Kapse, V.D.; Patil, D.R. *Curr. Appl. Phys.* 2012, *12*, 307–312) has been renumbered as 30. And the references after 30 was changed to 31, 32, 33, etc.

**References in the Original**
4. Nakatsuka, A.; Ikeda, Y.; Yamasaki, Y.; Nakayama, N.; Mizota, T. Cation distribution and bond lengths in CoAl_2_O_4_ spinel. *Solid State Commun.* **2003**, *128*, 85–90.
5. Kapse, S.D.; Raghuwanshi, F.C.; Kapse, V.D.; Patil, D.R. Characteristics of high sensitivity ethanol gas sensors based on nanostructured spinel Zn_1−x_Co_x_Al_2_O_4_. *Curr. Appl. Phys*. **2012**, *12*, 307–312.

**New References**
4. Li, X.; Zhu, Z.; Zhao, Q.; Wang, L. Photocatalytic degradation of gaseous toluene over ZnAl_2_O_4_ prepared by different methods: A comparative study. *J. Hazard. Mater.* **2011**, *186*, 2089–2096.
5. Rahnamaeiyan, S.; Nasiri, M.; Talebi, R.; Khademolhoseini, S. Novel sol–gel method for synthesis of cobalt aluminate and its photocatalyst application. *J. Mater. Sci. Mater. Electron.* **2015**, *26*, 8720–8725.
30. Kapse, S.D.; Raghuwanshi, F.C.; Kapse, V.D.; Patil, D.R. Characteristics of high sensitivity ethanol gas sensors based on nanostructured spinel Zn_1−x_Co_x_Al_2_O_4_. *Curr. Appl. Phys.* **2012**, *12*, 307–312.

## Figures and Tables

**Figure 3:**
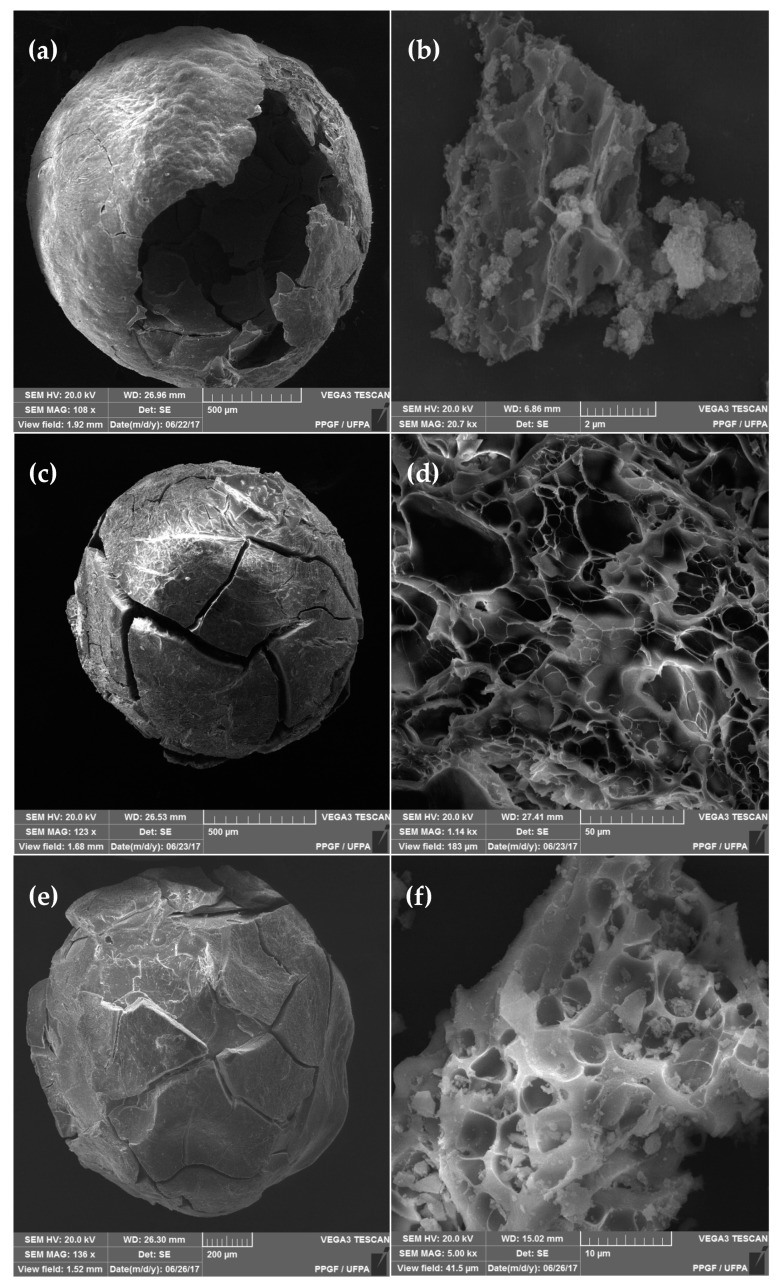
Scanning electron micrographs of samples (**a**) and (**b**) ZnAl_2_O_4_–750; (**c**) and (**d**) NiAl_2_O_4_–950; (**e**) and (**f**) CoAl_2_O_4_–750.
